# A modified mouse model for observational fear learning and the influence of social hierarchy

**DOI:** 10.3389/fnbeh.2022.941288

**Published:** 2022-07-25

**Authors:** Tianyao Shi, Shufang Feng, Wenlong Shi, Yuan Fu, Wenxia Zhou

**Affiliations:** ^1^State Key Laboratory of Toxicology and Medical Countermeasures, Beijing Institute of Pharmacology and Toxicology, Beijing, China; ^2^Department of Medical Psychology, The Third Medical Center, Chinese PLA General Hospital, Beijing, China

**Keywords:** observational fear, emotionality, integrated Z-score, susceptibility, social hierarchy

## Abstract

**Background:**

Indirectly experiencing traumatic events either by witnessing or learning of a loved one’s suffering is associated with the highest prevalence rates of epidemiological features of PTSD. Social species can develop fear by observing conspecifics in distress. Observational fear learning (OFL) is one of the most widely used paradigms for studying fear contagion in mice. However, the impact of empathic fear behavior and social hierarchy on fear transfer in mice is not well understood.

**Methods:**

Fear emotions are best characterized in mice by using complementary tests, rather than only freezing behavior, and simultaneously avoiding behavioral variability in different tests across time. In this study, we modified the OFL model by implementing freezing (FZ), open field (OF), and social interaction (SI) tests in a newly designed experimental facility and applied Z-normalization to assess emotionality changes across different behaviors.

**Results:**

The integrated emotionality scores revealed a robustly increased emotionality of observer mice and, more importantly, contributed to distinguishing susceptible individuals. Interestingly, fos-positive neurons were mainly found in the interoceptive network, and mice of a lower social rank showed more empathy-like behaviors.

**Conclusion:**

Our findings highlight that combining this experimental model with the *Z*-scoring method yields robust emotionality measures of individual mice, thus making it easier to screen and differentiate between empathic fear-susceptible mice and resilient mice, and refining the translational applicability of these models.

## Introduction

Fear is often indirectly acquired by social observation in groups (Olsson and Phelps, 2007). Witnessing or even hearing about others’ suffering can also be deemed traumatic, which may lead people to post-traumatic stress disorder (PTSD) (Yehuda et al., 2015). Like humans, rodents demonstrate affective sensitivity to their conspecifics’ pain (Langford et al., 2006; Gonzalez-Liencres et al., 2014). Observational learning of unpleasant events has also been studied in non-mammalian models, such as bird species; when approached by a potential predator, fear contagion appears in flocks (Griffin, 2004). Studying empathic fear may provide significant insights into neural mechanisms of social transfer of fear and related mental disorders. The most commonly used paradigm is observational fear learning (OFL) (Keum and Shin, 2019). Most previous studies have recognized OFL as a useful behavioral model for assessing fear contagion in mice (Kim et al., 2019). In this model, the observer mice are vicariously fear conditioned by viewing cagemates suffering from repetitive foot shocks (Jeon et al., 2010) or enemy attacks (Sial et al., 2016). The freezing behavior displayed by the observer mouse is measured with or without a demonstrator in distress for assessing their social fear transfer (Jeon and Shin, 2011).

However, whether the observer mouse truly recognizes how the demonstrator mouse feels and whether fear contagion can be assessed by only measuring the freezing behavior remains unclear. Even though freezing behavior in rodents is a direct, behavioral readout of fear (Ledoux, 2000), emotionality is not unidimensional. Empathic fear may vary in animals even under the same stimuli (Panksepp and Lahvis, 2011). Thus, ascribing a particular behavior to empathy is difficult. In animals, the evaluation of emotion-related behaviors is typically measured with a series of tests (Cryan and Holmes, 2005).

Empathic fear can be comprehensively understood in animals by combining different behavioral tests. Nevertheless, mice can be in different emotional states when exposed to multiple behavioral tests over several days (Ramos, 2008). To reduce intra-individual variation, the same animal should be introduced to different tests simultaneously, but not successively. For this purpose, some researchers have developed an integrated system by combining different test apparatuses. In the study of Ramos et al. (2008) and Fraser et al. (2010), three popular anxiety test apparatuses were physically combined so that an animal could freely explore all apparatuses during one single trial. This integrated system may provide more reliable information in the study of emotion. Hence, in our study, we modified the widely used OFL model in Jeon et al. (2010) study and designed an easier experimental setup to assess observational fear behavior by combining passive defensive (freezing), active avoidance (escape), and social interaction behaviors.

Convergent rather than consistent behaviors are the core of the clinical characterization of human mental illness. Behavioral parameters obtained from these triple tests that reflect fear response in animals may also have a large degree of individual differences. Here, we further introduced Z-normalization, a numerical measurement that standardizes observations at different times and from different cohorts (Van Steensel and Heeman, 2017), to assess the emotionality dimension of mice (Guilloux et al., 2011). This method allows us to compare multiple ethological variables with a single integrated *Z*-score.

In human society, social hierarchy affects interpersonal relationships and plays a key role in emotion contagion. Subordinate individuals tend to display higher basal corticosterone levels and increased anxiety-like behavior (Bartolomucci, 2007). For animals living in the same cage, the social hierarchy may also be a major factor in the regulation of emotional behavior (Karamihalev et al., 2020). However, the role of social hierarchy in mice fear transfer in the OFL paradigm remains unclear.

In the present study, we modified the OFL mouse model and calculated the emotional score of each mouse after observational fear. Subsequently, we assessed the neuronal activation of emotionally susceptible mice using c-fos as a maker and further investigated the relationship between social hierarchy and social fear transfer.

## Materials and methods

### Animals

All procedures were approved by the Beijing Institute of Basic Medical Science. Laboratory animal care was conducted in accordance with the Chinese Veterinary Medicine Association Guidelines and the International Association for the Study of Fear Conditioning. Unless otherwise specified, adult C57BL6/J male mice (8 weeks, 25–30 g) were used in this experiment. Mice were housed in groups of four per cage in 12-h light–dark phases (from 7 a.m. to 7 p.m.) and provided with a standard chow diet (Beijing Si Bei Fu Co.) and water *ad libitum*. All behavioral tests were conducted during the light cycle.

### Modified observational fear learning paradigms

#### Apparatuses

The chamber consisted of a white Plexiglas box (40 cm × 40 cm × 45 cm, long × wide × high) enclosed in a metallic chamber to reduce external sensory disturbance. The floor consisted of an unmovable stainless-steel grid connected to a foot-shock delivery unit. The chrome, steel, and pencil cup of 8 cm (diameter) × 10 cm (height), with ∼1 cm gaps between the bars, was sufficient for animal interactions and sniffing. A white Plexiglas board was covered on the top of the stainless-steel grid, and a hole was made at the corner to fit the size of the cup. This could ensure that only the mouse in the cup can access the electrodes. A digital camera was placed on the top of the apparatus to record and subsequently rescore the animals’ behavior in different areas, including interaction, corner, and side zones.

#### Observational fear procedure

**FIGURE 1:**
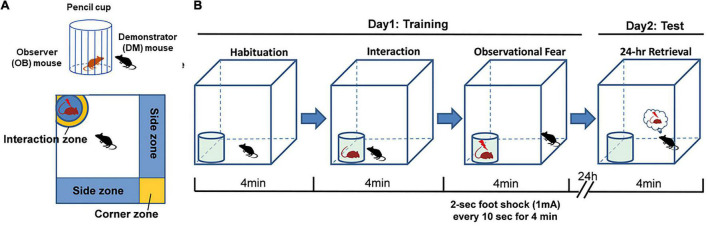
Modified observational fear learning in the mouse. **(A,B)** Diagram of the apparatus used for observational fear conditioning and the scheme of the behavioral assay.

On day 1, each observer (OB) mouse was placed in the chamber (with an empty cup) and allowed to freely explore for 4 min. After the habituation period, a partner male demonstrator (DM) mouse was placed in the cup, and the OB mouse was allowed to explore the DM mouse for 4 min. Then, during the observational fear period, the DM mouse was given an electric foot shock (2 s, 1 mA every 10 s for 4 min), allowing the OB mouse to observe and explore. Control mice underwent the same procedure but witnessed a no-shock DM mouse. Freezing, the side and corner time, and social interaction behavior were scored during this 4-min period. After training, the mice were returned to their home cages. On day 2, the OB mice were placed back into the chamber and tested for 4 min in this retrieval period (Figure 1).

### Open field test

The time and distance ratio spent in the corner and side of the same chamber were recorded to evaluate avoidance behaviors (the width of the corner and side was 10 cm). Here, we report the time in the center of the open field and the ratio of the distance traveled in the center (distance traveled in the corner and side divided by the total distance traveled × 100). The total distance traveled was used as an index of locomotor activity.

### Social interaction test

The cumulative times and entry times in the interaction zone were calculated from the video data of each trial. The social index is calculated as: time spent with the DM mouse during the interaction stage/time spent with the empty cup during the habituation stage.


$$S\,o\,c\,i\,a\,l\,I\,n\,d\,e\,x\,\left(S\,I\right)=\frac{I\,n\,t\,e\,r\,a\,c\,t\,i\,o\,n\,z\,o\,n\,e\,t\,i\,m\,e\,o\,f\,`\,`\,t\,a\,r\,g\,e\,t^{\prime \prime }\,t\,r\,i\,a\,l}{I\,n\,t\,e\,r\,a\,c\,t\,i\,o\,n\,z\,o\,n\,e\,t\,i\,m\,e\,o\,f\,`\,`\,n\,o\,t\,a\,r\,g\,e\,t^{\prime \prime }\,t\,r\,i\,a\,l}$$

Then, the Z-score of social interaction was averaged by the Z-head time spent and Z-head entry times in the interaction zone during the training or test stage. These are two different behavioral measures. One represents the total time spent with the head in the zone, the other is the number of head entries in the zone.


Z-=Social⁢interaction(Z-+Head⁢time⁢spentZ-)Head⁢Entries/2

### Tube test

The test was performed as previously described (Wang et al., 2011). Briefly, the mice were housed together for at least 4 weeks. Before training, mice were habituated to the Plexiglas tube (diameter, 3 cm; length, 30 cm) for 2 consecutive days. The size of the tube sufficed to allow an adult mouse to pass through without reversing its direction. During the training stage, two mice were grabbed by the tail and placed at each end of the tube until they reached the middle of the tube. Then, the tail was released and we recorded the situation in which one mouse pushes another mouse out of the tube. The mouse that retreated from the tube was defined as the “loser” and the other one as the “winner.” Each mouse fought against the other in the daily test trial. After each trial, the tube was cleaned with 70% ethanol to remove odor, urine, or feces. At the end of the 6-day test, the four mice raised in the same cage were ranked according to the number of wins by ranking the most dominant mice 1 and 2 and the most subordinate mice 3 and 4.

### Emotionality *Z*-score calculation

To obtain comprehensive and integrated behavioral measures, emotionality-related data were normalized using a *Z*-score methodology, as previously described (Guilloux et al., 2011). Briefly, the behavioral parameters of each mouse were calculated using the following formula to calculate the *Z*-scores. μ and SD are, respectively, the mean and standard deviations of the control group. X is the observational data for each mouse in the OB group.


$$Z=\frac{X-\mu }{S\,D}$$

Individual emotionality scores were then calculated by averaging *Z*-scores within tests: *Z*-_*Open field*_ = (*Z*-_*Corner and Side time*_ + *Z*-_*Distance Ratio*_)/2, *Z*_*SI*_ = (*Z*-_*Head time* spent_ + *Z*-_*Head Entries*_)/2, subsequently calculating an emotionality *Z*-score for each animal based on three different tests on the training or testing day according to the following formula:


$$E\,m\,o\,t\,i\,o\,n\,a\,l\,i\,t\,y\,s\,c\,o\,r\,e=\frac{\left|Z_{F\,Z}\right|+\left|Z_{O\,F}\right|+\left|Z_{S\,I}\right|}{N\,m\,u\,b\,e\,r\,o\,f\,t\,e\,s\,t}$$

Last, the total emotion score was calculated by averaging the emotionality score on training and testing days.


$$T\,o\,t\,a\,l\,e\,m\,o\,t\,i\,o\,n\,a\,l\,i\,t\,y\,s\,c\,o\,r\,e=\frac{Z_{t\,r\,a\,i\,n\,i\,n\,g}+Z_{t\,e\,s\,t}}{2}$$

### Immunohistochemistry

After the retrieved memory behavioral tests, mice were transcranial perfused under anesthesia with 50 ml PBS followed by 20 ml cold 4% w/v paraformaldehyde (PFA). The brains were postfixed for 8–12 h and dehydrated with 30% sucrose. The brains were then sectioned into 40-μm thick coronal slices using a vibratome (VT1000S, Leica). To confirm c-fos expression, the brain slices were rinsed in PBS and incubated in a blocking solution (0.1% Triton X-100, 1% bovine serum albumin, and 5% normal goat serum in PBS) for 1 h at room temperature, followed by overnight incubation with primary antibody at 4°C. The primary antibodies were rabbit anti-c-fos (#2,250, cell signaling, 1:500). The secondary antibodies were Alexa Fluor 488 goat anti-rabbit IgG (Thermo Fisher Scientific, 1:1,000).

Quantification of Fos staining was performed on every 6th slice in the following area: Anterior cingulate cortex (ACC) from Bregma 1.34 to −0.22 mm (six sections per mice), anterior insular cortex (AIC) from Bregma −0.7 to −1.92 mm (six sections per mice), basolateral amygdala (BLA) from Bregma −0.58 to −1.34 mm (six sections per mice), hippocampus (HIP) from Bregma −1.46 to −2.30 mm (five sections per mice), perirhinal cortex (PRh) from Bregma −2.46 to −2.70 mm (five sections per mice), and paraventricular nucleus (PVN) from Bregma −0.58 to −1.22 mm (three sections per mice). All images were subsequently overlaid with the corresponding atlas section to anatomically define the regions of interest. Positive cells lying on the boundary were excluded. A cell was considered positive only if it displayed an intensity value above the intensity threshold of the background. Seven brains were used for each experiment. Quantification was performed using the cell counter tool in ImageJ.

### Statistical analysis

All data were shown as mean ± SEM unless otherwise specified. All data analyses were performed using the statistical software, Prism 8.1 (GraphPad). Comparisons were conducted with the one- and two-way analysis of variance (ANOVA), student’s *t*-test, and the Mann-Whitney *U*-test to identify significant differences at *p*-value of < 0.05.

Both substantively significant (*p*-value) and significant (effect size) results were reported (Fritz et al., 2012). Cohen’s *d* effect size coefficients were calculated to estimate the relative magnitude of the differences in the comparisons. Cohen’s *d* is simply a measure of the distance between two means. According to the absolute values of Cohen’s *d* (*d*), the following cutoffs corresponding to the magnitude of the differences were used: *d* ≤ 0.5 (small effect), 0.5 < *d* < 1.0 (moderate effect), 1.0 ≤ *d* < 1.5 (large effect), and *d* ≥ 1.5 (very large effect) (Labots et al., 2016).

## Results

### Freezing behavior and *Z*-scores during observational fear training

**FIGURE 2:**
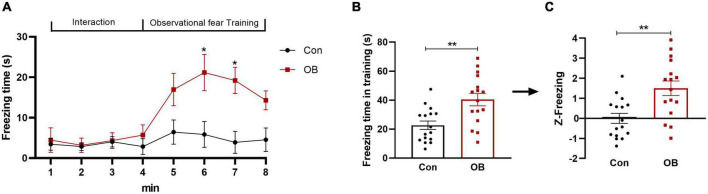
Freezing behavior and emotionality *Z*-score in FZ. **(A,B)** A significant difference in the level of freezing behavior was apparent during the observational fear training and retrieval phase. **(C)** Normalization of data using the *Z*-score method was performed for the freezing time of each mouse in the training phase. Control group: OB mouse paired with a no-shock DM mouse. Model group: OB mouse paired with a foot-shock DM mouse. Data are expressed as mean ± SEM (*n* = 16) and compared by unpaired *t*-test **(B,C)** or two-way ANOVA followed by Bonferroni’s multiple comparison test **(A)**. *p < 0.05, **p < 0.01.

In this apparatus, the observer (OB) mouse was allowed to observe the demonstrator (DM) mouse being subjected to repetitive foot shocks in the corner pencil cup. It clearly showed a significantly increased freezing behavior as he saw the distressed DM mouse in comparison with the control group [training phase: *t*_(30)_ = 3.408, *p* = 0.0019, *n* = 16, unpaired *t*-test; Figures 2A,B] during the observational fear training phase. On day 2 of the fear memory retrieval phase, the OB mouse also showed significantly increased freezing when returning to the environment in which the traumatic events had occurred [test phase: *t*_(30)_ = 2.094, *p* = 0.0448, *n* = 16, unpaired *t*-test]. Freezing parameters of the observational training phase were then normalized to the *Z*-score by transforming absolute values to numbers of SDs above or below the mean of the control. This step yielded a same *p*-value as that before normalization [*t*_(30)_ = 3.408, *p* = 0.0019, *n* = 16, unpaired *t*-test; Figure 2C].

### Avoidance behavior and *Z*-scores during observational fear training

**FIGURE 3:**
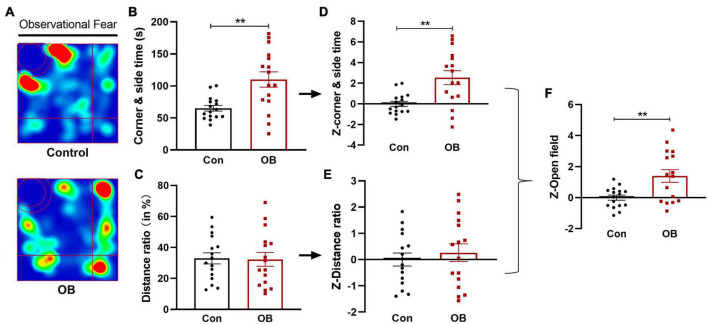
Avoidance behavior and emotionality *Z*-scores in open field (OF) test. **(A)** Representative locomotor path of mice in the two groups in the observational fear training stage. **(B)** Mice in the model group spent significantly more time in the corner and side than the control group. **(C)** No significant difference in distance moved in the corner and side area (%). **(D,E)** Normalization of *Z*-score for the two parameters with the same statistical *P*-values. **(F)** Z-OF calculated by averaging *Z*-scores of the two OF parameters showed a significant difference between the two groups. Data are expressed as mean ± SEM (*n* = 16) and compared by unpaired *t*-test. **p < 0.01.

In the open field (OF) test, the OB mice spent more time in the corner and side than controls while witnessing their partner in distress [*t*_(30)_ = 3.517, *p* = 0.0014, *n* = 16, unpaired *t*-test; Figures 3A,B]. Observational fear stress exposure did not affect the relative travel distance in the corner and side area [*t*_(30)_ = 0.1128, *p* = 0.9109, *n* = 16, unpaired *t*-test; Figure 3C]. The respective behavioral parameters were then converted into normalized *Z*-values, and this process did not change the significant differences (Figures 3D,E). Last, the *Z*-score per OF mouse was averaged to calculate a single OF *Z*-score, showing that the OB mice spent significantly more time in the corner and side than controls during observational fear training [*t*_(30)_ = 3.160, *p* = 0.0036, *n* = 16, unpaired *t*-test; Figure 3F].

### Social behavior and *Z*-scores during observational fear training

**FIGURE 4:**
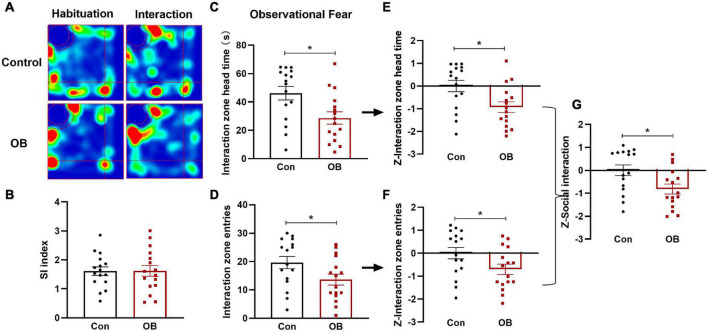
Social behavior and emotionality *Z*-scores in social interaction (SI). **(A)** Representative locomotor path of mice in the two groups in habituation and interaction stage. **(B)** No significant difference in the social index between the two groups during the first two stages. The social index is calculated as: time spent with the DM mouse during interaction/time spent with the empty cup during habituation. **(C,D)** Head time and entries in the interaction zone of the model group were both lower than that of the control during the observational fear stage. **(E,F)** Normalization of *Z*-score for the two parameters with the same statistical *P*-values. **(G)** Z-SI calculated by averaging *Z*-scores of the two SI parameters showed a significant difference between the two groups. Data are expressed as mean ± SEM (*n* = 16) and compared by unpaired *t*-test. *p < 0.05.

In the social interaction (SI) test, both groups exhibited clear social preferences for a familiar partner than for the empty pencil cup during the first two stages, with no differences in social index values [*t*_(30)_ = 0.0299, *p* = 0.9763, *n* = 16, unpaired *t*-test; Figures 4A,B]. In the observational fear training phase, the OB group exhibited more social avoidance behaviors than the control group with significantly decreased head time [*t*_(30)_ = 2.725, *p* = 0.0106, *n* = 16, unpaired *t*-test; Figure 4C] and entries in the interaction zone [*t*_(30)_ = 2.090, *p* = 0.0452, *n* = 16, unpaired *t*-test; Figure 4D]. Using the same *Z*-score standardization, the time and entries *Z*-scores were calculated, respectively (Figures 4E,F), and then averaged to determine the *Z*-score of social interaction [*t*_(30)_ = 2.550, *p* = 0.0161, *n* = 16, unpaired *t*-test; Figure 4G]. Based on the statistical results of the Z-Social interaction between two groups, social avoidance behavior was significantly increased in the OB mouse that witnessed a partner in stress.

### Integrated emotionality *Z*-scores in mice exposed to observational fear training

**FIGURE 5:**
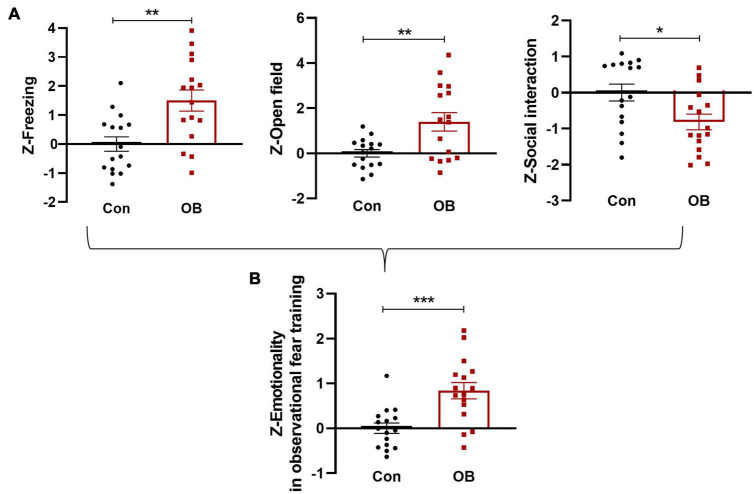
Integrated emotionality *Z*-scores in mice exposed to observation fear training on day 1. **(A)** FZ, OF, and SI test *Z*-values were calculated by averaging individual *Z*-scores as described above and **(B)** averaged to obtain an integrated emotionality *Z*-score. Data are expressed as mean ± SEM (*n* = 16) and compared by unpaired *t*-test. *p < 0.05, **p < 0.01, ***p < 0.001.

Single *Z*-scores were calculated per mouse and per behavioral test. Analyses of test-specific *Z*-scores indicated a significant effect of observational fear training on freezing, avoidance, and social behaviors, with the OB group displaying higher *Z*-scores than the control group (Figure 5A). All three tests were weighted similarly, and a single “emotionality score” was calculated for each mouse on the observation fear training day, described as the integrated output of emotionality. The results of each test *Z*-scores were compared, with the combined normalized measures of emotionality resulting in the augmented statistical significance of the observational fear main effect [*t*_(30)_ = 3.907, *p* = 0.0005, *n* = 16, unpaired *t*-test; Figure 5B].

### Integrated total emotionality *Z*-scores on observation fear training and testing day

**FIGURE 6:**
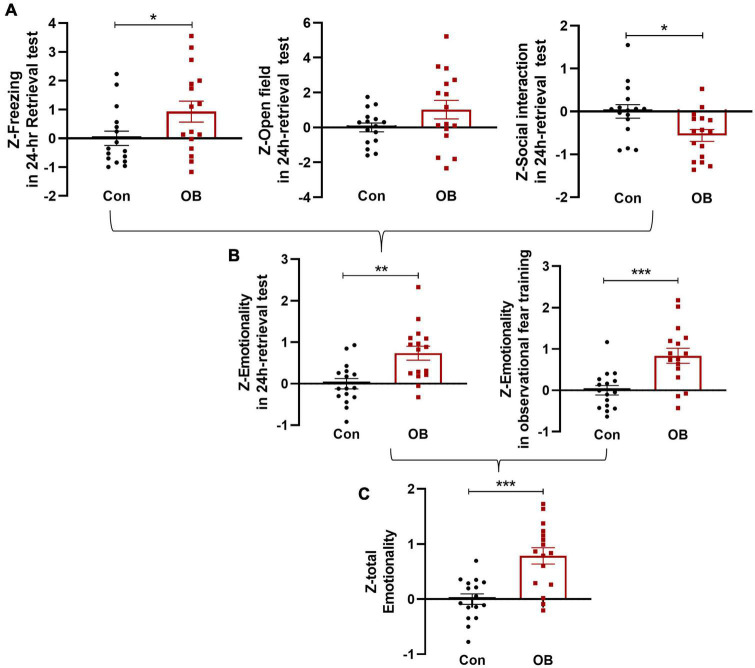
Integrated total emotionality of training and testing day. **(A)** Raw data are obtained from three behavioral tests (FZ, OF, and SI) performed on the same animals. Normalization of data using the *Z*-score method was performed for each parameter. FZ, OF, and SI test *Z*-values were calculated on a 24-h retrieval testing day. **(B,C)** *Z*-scores on training and testing days were averaged to obtain an integrated total emotionality *Z*-score. Data are expressed as mean ± SEM (*n* = 16) and compared by unpaired *t*-test. *p < 0.05, ^**^p < 0.01, ^***^p < 0.001.

In the 24-h retrieval test, single *Z*-scores were also calculated per mouse and per behavioral test, as described above. Some OB mice did not show fear-like behavior and exhibited decreased differences in FZ [*t*_(30)_ = 2.094, *p* = 0.0448, unpaired *t*-test] and OF [*t*_(30)_ = 2.024, *p* = 0.0520, unpaired *t*-test, *n* = 16; Figure 6A] test. However, the combined *Z*-scores of 24 h emotionality also showed a more noticeable increase in OB group [*t*_(30)_ = 3.520, *p* = 0.0014, *n* = 16, unpaired *t*-test, compared with control group; Figure 6B]. Last, to determine a single “total emotionality score” for each mouse in this experiment, *Z*-scores of emotionality on training and testing days were averaged. The results suggested that integrated Z-scores provide a more robust overall effect when evaluating the observational fear behavior of mice across complementary behavioral dimensions [*t*_(30)_ = 4.451, *p* = 0.0001, *n* = 16, unpaired *t*-test; Figure 6C].

### Analyses of the integrated *Z*-scores and identifying the susceptible mice

**FIGURE 7:**
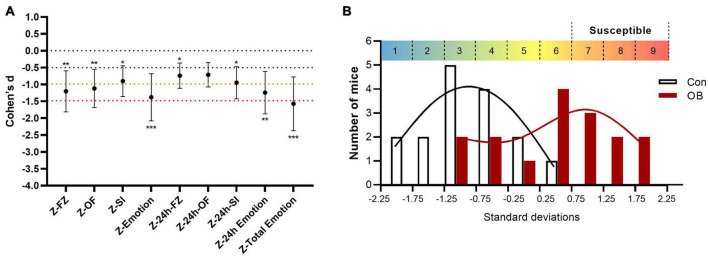
Analyses of the integrated *Z*-scores and identify the empathy susceptible mice. **(A)** Cohen’s *d*-values for behavioral parameters, single test *Z*-scores, and integrated *Z*-scores. **(B)** A stanine (“standard nine”) score to scale *Z*-scores on a nine-point scale. Error bars represent 95% CI of Cohen’s *d*. *p < 0.05; **p < 0.01; ***p < 0.001 for the statistical significance in the corresponding unpaired *t*-test.

Effect size is a quantitative measure of the magnitude of the experimental effect. A significant *p*-value informs us that an intervention works, whereas an effect size expresses how much it works. In this study, the final total emotionality *Z*-scores of the OB group, as a single indicator, augmented the statistical significance, with effect sizes advancing to a “very large effect” (*d* ≥ 1.5), as verified using the Pearson’s correlation coefficients (Figure 7A). Nevertheless, not all mice showed significant learned fear after integrating *Z*-scores. Some intra-individual variation remained in the OB group. Then, we used Stanine (an abbreviation of STAndard NINE), which is a method for scaling test scores on a nine-point standard scale, to select better susceptible mice for further research. The distribution is divided into nine categories. Each category is 0.5 SD wide (except for the 1st and 9th stanine). Scores higher than 0.75 are considerably above average. Ultimately, 7 of the 16 OB mice were chosen as susceptible mice in the 7th to 9th stanine section (Figure 7B) for the next experiments, with the control group composed of seven randomly selected control mice.

### Activated neurons in different brain regions of susceptible mice after observational fear

**FIGURE 8:**
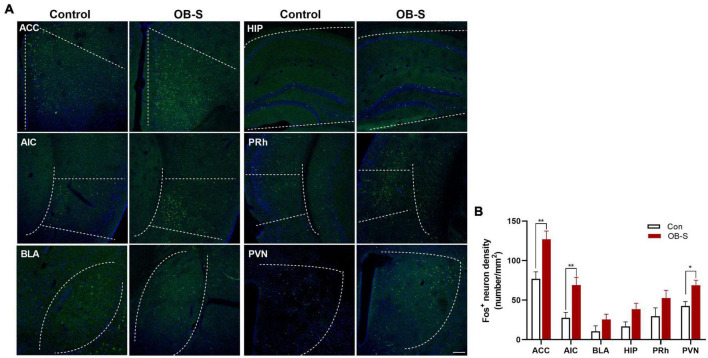
Activation of neurons in different brain regions of susceptible mice. **(A,B)** Quantification of fos-like immunoreactivity in ACC, AIC, BLA, HIP, PRh, and PVN. ACC anterior cingulate cortex, AIC anterior insular cortex, BLA basolateral amygdale, HIP hippocampus, PRh perirhinal cortex, PVN paraventricular nucleus. *n* = 7 mice per group. Data are presented as means ± SEM. Sidask’s test analysis between each group as indicated *p < 0.05, **p < 0.01. Scale bar, 100 μm.

To further explore the involvement of brain regions in processing fear contagion, we used Fos, an immediate early gene with a well-characterized activity-dependent expression pattern (Dragunow and Faull, 1989) to identify neurons activated in selected (7 out of 16; Figure 7B) susceptible OB mice (OB-S). In comparison with the control group, we observed Fos^+^ neurons expression in several brain regions, including the ACC, the anterior insular cortex (AIC), the basolateral amygdale (BLA), the hippocampus (HIP), the perirhinal cortex (PRh), and the paraventricular thalamic nucleus (PVN) (Figure 8A), which are partly involved in observational fear (Ito et al., 2015; Pisansky et al., 2017; Allsop et al., 2018). The density of Fos^+^ neurons was significantly higher in ACC, AIC, and PVN in OB-S group than in control group [two-way ANOVA, ACC, *F*_(1,72)_ = 3.913, *p* = 0.0012; AIC, *F*_(1,72)_ = 3.754, *p* = 0.0021; BLA, *F*_(1,72)_ = 1.821, *p* = 0.3645; HIP, *F*_(1,72)_ = 1.901, *p* = 0.3160; PRh, *F*_(1,72)_ = 1.725, *p* = 0.4277; PVN, *F*_(1,72)_ = 2.715, *p* = 0.0487, *n* = 7; Figure 8B], and a rising trend was also detected in BLA, HIP, and PRh. Together, our imaging data indicate that susceptible mice show a robust neuronal activation in ACC, AIC, and PVN *in vivo*.

### Social hierarchy affects social fear transfer in group mice

**FIGURE 9:**
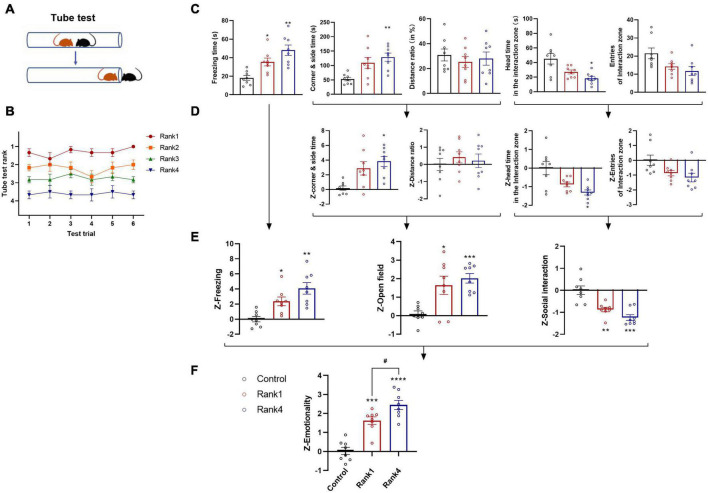
Social hierarchy affects social fear transfer. **(A)** Schematic of the tube test. **(B)** Overall rank stability shows the average rank of animals belonging to each rank group (as determined at the end of the tube test) calculated for each day of testing. **(C)** Raw data are obtained from three behavioral tests (FZ, OF, and SI; *n* = 8/group) performed on the same animals. **(D)** Normalization of data using the *Z*-score method was performed for each parameter. **(E)** Test *Z*-values were then calculated by averaging individual *Z*-scores, and **(F)** averaged to obtain the emotionality score. Data represent mean ± SEM (*n* = 8/group). *p < 0.05; **p < 0.01; ***p < 0.001 and ****p < 0.0001 for effects of dominate (Rank 1) and subordinate (Rank 4) group compared to the control group. ^#^*p* < 0.05 for effects between Rank 1 and Rank 4 groups.

For both animals and humans who live in groups, social bonds, such as kinship, familiarity, and social hierarchy, are important for perceiving conspecifics’ emotions. Hierarchical status greatly influences social interactions and vulnerability to stress (Wang et al., 2014; Larrieu et al., 2017). In our study, we also assessed whether preexisting dominance hierarchies may affect social fear transmission during observational fear training. To this end, we used the tube test (Wang et al., 2011), a reliable paradigm for measuring dominant behavior in rodents and validating social dominance in male mice housed in pairs (Figure 9A). A group of four mice formed a clear social hierarchy after 6 days of trials (Figure 9B). In the next observational fear training, the most dominant (Rank 1) and the most subordinate (Rank 4) mice were assigned as demonstrator and observer mice, respectively. The remaining middle-class (Rank 2–3) mice were subjected to stress, as demonstrators. The *Z*-scores of emotionality were calculated as described above, and the results of individual tests are as follows (Figure 9C): in the FZ test, both dominant and subordinate mice showed significantly higher freezing times than controls [*F*_(2,21)_ = 1.952, one-way ANOVA, Rank 1 vs. control, *p* < 0.05 and Rank 4 vs. control, *p* < 0.01, *n* = 8], whereas no significant effects were observed between Rank 1 and Rank 4 mice; in the OF test, only Rank 4 group displayed increased corner and side time compared to control mice [*F*_(2,21)_ = 1.984, one-way ANOVA, *p* < 0.01, *n* = 8]; in the SI test, Rank 4 mice also spent significantly less head time into the interaction zone than controls [*F*_(2,21)_ = 2.563, one-way ANOVA, *p* < 0.05, *n* = 8]. No significant effect was observed in entries in the interaction zone. To assess a more stable underlying trend, we performed *Z*-score normalization as described above (Figure 9D). The results were then averaged to determine the single value for each mouse and each behavioral test (Figure 9E). Observational fear training had a significant main effect on *Z*-scores in all three tests. Last, we averaged all values to calculate a single “emotionality score,” thus identifying the most significant difference between groups [*F*_(2,21)_ = 0.5248, one-way ANOVA, Rank 1 *p* < 0.001, Rank 4 *p* < 0.0001, compared with the control group, *n* = 8]. The Rank 4 group had a significantly higher emotionality score than the Rank 1 group (*p* < 0.05), which proved that social status has an impact on emotion perception (Figure 9F). Thus, using *Z*-score normalization, a robust underlying trend can be derived from more variable individual measurements, critically confirming that lower social hierarchy may be associated with the higher social fear transfer.

## Discussion

Accurate perception of potential threats is essential for survival. Empathy enables human and non-human primates to recognize the emotional state of others. In rodents, some studies have shown that exposure to social cues signaling threat, such as the sight, sound, or smell of a scared conspecific, may trigger or potentiate fear responses (Kim et al., 2010; Inagaki et al., 2014), a process that is termed fear contagion (Dezecache et al., 2015; Keum and Shin, 2016). Understanding the neural basis of empathy is crucial for understanding the neural mechanisms of social behaviors and related mental disorders (Decety and Moriguchi, 2007; Thirioux et al., 2019), such as anxiety, autism, and vicarious trauma (usually resulting from repeated exposure to other people’s trauma). OFL is the most used behavior paradigm in studying rodents’ fear contagion, in which an unconditioned stimulus (US) is vicariously provided by observing conspecifics receiving foot shocks (Jeon et al., 2010; Kim et al., 2012; Twining et al., 2017). In this paradigm, observers’ fear responses, such as freezing or immobility, are the most used measurement parameters. Avoidance behavior is also tested in other studies to measure fear contagion (Masuda and Aou, 2009; Debiec and Sullivan, 2014). Indirectly acquired fear responses are influenced by many factors and vary from person to person. Facing a companion in distress, rodents may exhibit different emotional behaviors, such as observational fear (Jeon et al., 2010), social coordination of pain (Langford et al., 2006), comfort (Burkett et al., 2016), avoidance (Masuda and Aou, 2009), and interruption of innate behavior (Allsop et al., 2018). Here, we modified the OFL assay system by performing freezing, open-field, and social interaction tests in a single trial, thereby minimizing the impact of emotional fluctuation on the short-term assessment of emotionality. Observational fear can be “diagnosed” through a series of variable symptoms. Thus, fear contagion is defined based on a series of converging behavior, rather than a single consistent behavior.

Subsequently in our study, Z-normalization and the integrated *Z*-scores were introduced to interpret and compare all aforementioned behavioral results. This method allows us to standardize outcome data from all the results from the various tests used in an investigation on the same comparable scale. *Z*-scores are already in use or have been proposed in various areas, such as clinical trials (Simpson et al., 2018), neurocognitive tests (Bergemann et al., 2012), cell profiling (Caicedo et al., 2017), and rodent emotionality (Guilloux et al., 2011; Labots et al., 2018). In this study, we propose using *Z*-score conversions for assessing the relative sensitivity of different dependent measures, which could facilitate the comparison of effect magnitudes and thus contribute to the evaluation of test sensitivity. In the FZ test, the freezing time of the OB group significantly increased during observational fear training and the 24-h contextual memory retrieval stage, in line with other studies (Jeon et al., 2010; Jeon and Shin, 2011; Smith et al., 2021). In addition to behavioral inhibition, active avoidance behaviors (escape or social avoidance) are also primary defensive methods for mice. In the OF and SI tests, both the OB groups showed a significant preference for the non-demonstrator side (more corner and side time and fewer head entries). However, these two avoidance behaviors do not always change simultaneously. We investigated the correlation of the integrated *Z*-scores between these three behavioral tests on the observational fear training day and found no significant correlation. However, Z-OF may be inversely related to Z-SI (*p* < 0.1) (Supplementary Material), suggesting a variation in behaviors between mice even when facing the same stimulus. For example, we found that some mice spent more time in the corner and side zone, despite having a high level of social interaction, most likely resulting from constantly shuttling between these two areas. The mice may “hesitate” to help or not. Therefore, in our experiments, we did not blindly seek a high correlation between each test but hoped to obtain convergence of results by integrating *Z*-scores. Ultimately, by using Z-normalization in different behavioral tests, we assigned a single score to each mouse, which can be used to quantitatively “diagnosis” their emotionality. This procedure could be useful for objectively evaluating the differential sensitivity of various metrics. Therefore, the integrated total *Z*-score enhanced the overall statistical significance of emotionality induced by observational fear. Furthermore, by Cohen’s *d* comparison, the effect size advanced from a “large effect” in a single test to a “very large effect” in integrated emotionality *Z*-scores (Figure 7A). Hence, for some behavioral tests subject to variability, positive results are often difficult to reconcile across tests. The integrated *Z*-score analysis is not only used to increase statistical significance but also to extract potential trends from apparently variable results.

Although this method increased statistical significance among groups, a large variation remained within groups. Stress susceptibility or resilience are common phenomena in animal (Sillivan et al., 2017) and human (Liu et al., 2018) research. Since each mouse receives a final *Z*-score, we ranked all *Z*-scores from the lowest to the highest to distinguish emotional susceptibility. A stanine score, short for “standard nine” score, is a way to rank test scores on a nine-point standard scale. Using this method, we can convert every *Z*-score from the original score to a number between 1 and 9. In general, we regard test scores as: Stanines 1, 2, and 3: below average (Resilience); Stanines 4, 5, and 6: average; Stanines 7, 8, and 9: above average (susceptible). Mice have been characterized as either susceptible or resilient to emotional stress. Subsequently, we investigated changes in c-fos expression in susceptible mice (OB-S group) after observational fear testing. Fos-positive cell density shows the trend of incense in many brain regions in comparison with the control group. Especially, in the ACC, the anterior insular cortex (AIC), and the paraventricular nucleus (PVN), we observed a significant increase in activated neurons, in line with the results of some other studies (Pisansky et al., 2017; Jhang et al., 2018; Rodriguez et al., 2019). So far, our current understanding of fear contagion largely derives from studies based on different classes of threats, including predator fear, aggressive conspecifics, or physically harmful stimuli. In conclusion, all classes of threats seem to interact with a common memorization unit centered in the amygdala, the hippocampus, and the cortex (Cho et al., 2017). No significant activation in the amygdala and the hippocampus has been thought of as the fear center or where fear engrams cell exists. In fact, a vigorous debate concerning what is fear has been playing out across the field of affective neuroscience. One of the scientists, Lisa Feldman Barrett advanced a provocative “theory of constructed emotion,” which proposes that the human brain constructs instances of fear as a consequence of predicting and inferring the cause of incoming sensory inputs from the body (Interoception) and the outer world (Barrett, 2017). As domain-general regions of the interoceptive system, ACC and AIC participate in emotion, autonomic functions, or self-awareness (Craig, 2003; Critchley, 2005). In our study, neural activity changes in these two areas indicated that the interoceptive system might play a role in the perception of others’ fear.

In our study, mice of the same strain and gender were used as experimental subjects. In co-caged mice, the social hierarchy may be a major determining factor for vulnerability to witnessing fears. Many research studies have proved that hierarchy moderates the effect of status on stress and performance in humans (Sapolsky, 2005; Knight and Mehta, 2017) and social animals (Karamihalev et al., 2020). However, one study has shown that conditioned fear may largely transfer to a subordinate cagemate by proxy (Jones and Monfils, 2016). How social dominance hierarchy leads to emotional susceptibility differences is not yet directly studied in an observational fear model. In our study, we show that social dominance hierarchy predicts the intensity of fear transmission and that subordinate mice display significantly increased fear responses (*p* < 0.05, compared with dominant mice) after interacting with a shocked demonstrator. Moreover, we were able to combine individual experiments by integrating emotionality *Z*-scores. Overall, the results showed that subordinate mice were more sensitive than dominant mice to indirect emotional stress. These consolidate social hierarchy-related differences in rodents.

## Conclusion

We developed an observational fear animal model to evaluate individual differences in fear contagion with respect to the emotional variation observed in human post-traumatic stress symptoms. The current study suggests that using a *Z*-score-based method is a useful procedure for objectively evaluating the differential sensitivity of various metrics. Integrated Z-normalization provides us with the advantage of addressing inherent difficulties in “consistent” behavioral phenotyping across tests and time, thereby simply and intuitively summarizing results. Overall, our results pave the roadway toward further studies in which susceptible and resilient animals should be differentially manipulated at the level of neural circuits and synaptic plasticity to better understand the neurobiological underpinnings of susceptibility and resilience to vicarious trauma development, which may help bridge the gap between pre-clinical and clinical studies and further provide effective therapeutic interventions in animal models, subsequently translated to humans.

## Data availability statement

The original contributions presented in this study are included in the article/Supplementary Material, further inquiries can be directed to the corresponding author.

## Ethics statement

The animal study was reviewed and approved by the Institute Animal Care and Use Committee (IACUC) of the National Beijing Center for Drug Safety Evaluation and Research (NBCDSER) (No. 2020-529). Written informed consent was obtained from the owners for the participation of their animals in this study.

## Author contributions

TS, SF, and WZ involved in designing the study. TS and SF carried out all experiments, analyzed the data, and wrote the manuscript. WZ helped to revise the manuscript. WS and YF helped to perform the behavioral testing. All authors had read and agreed to the published version of the article.

## Supplementary material

Supplementary Figure 1 The relationship with the integrated *Z*-scores of three different tests (FZ, OF, and SI) on observational fear training. **(A–C)** There were no significant correlations between the integrated Z-scores calculated from FZ, OF, and SI. Data are analyzed by Pearson’s correlation.

## References

[B1] Allsop S. A. Wichmann R. Mills F. Burgos-Robles A. Chang C. J. Felix-Ortiz A. C. (2018). Corticoamygdala Transfer of Socially Derived Information Gates Observational Learning. *Cell* 173 1329–1342.e18. 10.1016/j.cell.2018.04.004 29731170 PMC6345560

[B2] Barrett L. F. (2017). The theory of constructed emotion: an active inference account of interoception and categorization. *Soc. Cogn. Affect. Neurosci.* 12 1–23. 27798257 10.1093/scan/nsw154 PMC5390700

[B3] Bartolomucci A. (2007). Social stress, immune functions and disease in rodents. *Front. Neuroendocrinol.* 28 28–49. 10.1016/j.yfrne.2007.02.001 17379284

[B4] Bergemann T. L. Bangirana P. Boivin M. J. Connett J. E. Giordani B. J. John C. C. (2012). Statistical Approaches to Assess the Effects of Disease on Neurocognitive Function Over Time. *J. Biom. Biostat.* 7:7310. 10.4172/2155-6180.S7-016 PMC4221235 25383237

[B5] Burkett J. P. Andari E. Johnson Z. V. Curry D. C. De Waal F. B. Young L. J. (2016). Oxytocin-dependent consolation behavior in rodents. *Science* 351 375–378. 10.1126/science.aac4785 26798013 PMC4737486

[B6] Caicedo J. C. Cooper S. Heigwer F. Warchal S. Qiu P. Molnar C. (2017). Data-analysis strategies for image-based cell profiling. *Nat. Methods* 14 849–863. 28858338 10.1038/nmeth.4397 PMC6871000

[B7] Cho J. H. Rendall S. D. Gray J. M. (2017). Brain-wide maps of Fos expression during fear learning and recall. *Learn. Mem.* 24 169–181. 10.1101/lm.044446.116 28331016 PMC5362696

[B8] Craig A. D. (2003). Interoception: the sense of the physiological condition of the body. *Curr. Opin. Neurobiol.* 13 500–505. 12965300 10.1016/s0959-4388(03)00090-4

[B9] Critchley H. D. (2005). Neural mechanisms of autonomic, affective, and cognitive integration. *J. Comp. Neurol.* 493 154–166. 16254997 10.1002/cne.20749

[B10] Cryan J. F. Holmes A. (2005). The ascent of mouse: advances in modelling human depression and anxiety. *Nat. Rev. Drug Discov.* 4 775–790. 10.1038/nrd1825 16138108

[B11] Debiec J. Sullivan R. M. (2014). Intergenerational transmission of emotional trauma through amygdala-dependent mother-to-infant transfer of specific fear. *Proc. Natl. Acad. Sci. U.S.A.* 111 12222–12227. 10.1073/pnas.1316740111 25071168 PMC4142995

[B12] Decety J. Moriguchi Y. (2007). The empathic brain and its dysfunction in psychiatric populations: implications for intervention across different clinical conditions. *Biopsychosoc. Med.* 1:22. 10.1186/1751-0759-1-22 18021398 PMC2206036

[B13] Dezecache G. Jacob P. Grezes J. (2015). Emotional contagion: its scope and limits. *Trends Cogn. Sci.* 19 297–299. 10.1016/j.tics.2015.03.011 25891260

[B14] Dragunow M. Faull R. (1989). The use of c-fos as a metabolic marker in neuronal pathway tracing. *J. Neurosci. Methods* 29 261–265. 10.1016/0165-0270(89)90150-7 2507830

[B15] Fraser L. M. Brown R. E. Hussin A. Fontana M. Whittaker A. O’leary T. P. (2010). Measuring anxiety- and locomotion-related behaviours in mice: a new way of using old tests. *Psychopharmacology* 211 99–112. 10.1007/s00213-010-1873-0 20454890

[B16] Fritz C. O. Morris P. E. Richler J. J. (2012). Effect size estimates: current use, calculations, and interpretation. *J. Exp. Psychol. Gen.* 141 2–18. 21823805 10.1037/a0024338

[B17] Gonzalez-Liencres C. Juckel G. Tas C. Friebe A. Brune M. (2014). Emotional contagion in mice: the role of familiarity. *Behav. Brain Res.* 263 16–21. 24480421 10.1016/j.bbr.2014.01.020

[B18] Griffin A. S. (2004). Social learning about predators: a review and prospectus. *Learn. Behav.* 32 131–140. 10.3758/bf03196014 15161148

[B19] Guilloux J. P. Seney M. Edgar N. Sibille E. (2011). Integrated behavioral z-scoring increases the sensitivity and reliability of behavioral phenotyping in mice: relevance to emotionality and sex. *J. Neurosci. Methods* 197 21–31. 10.1016/j.jneumeth.2011.01.019 21277897 PMC3086134

[B20] Inagaki H. Kiyokawa Y. Tamogami S. Watanabe H. Takeuchi Y. Mori Y. (2014). Identification of a pheromone that increases anxiety in rats. *Proc. Natl. Acad. Sci. U.S.A.* 111 18751–18756. 10.1073/pnas.1414710112 25512532 PMC4284601

[B21] Ito W. Erisir A. Morozov A. (2015). Observation of Distressed Conspecific as a Model of Emotional Trauma Generates Silent Synapses in the Prefrontal-Amygdala Pathway and Enhances Fear Learning, but Ketamine Abolishes those Effects. *Neuropsychopharmacology* 40 2536–2545. 10.1038/npp.2015.100 25865929 PMC4569943

[B22] Jeon D. Kim S. Chetana M. Jo D. Ruley H. E. Lin S. Y. (2010). Observational fear learning involves affective pain system and Cav1.2 Ca2+ channels in ACC. *Nat. Neurosci.* 13 482–488. 10.1038/nn.2504 20190743 PMC2958925

[B23] Jeon D. Shin H. S. (2011). A mouse model for observational fear learning and the empathetic response. *Curr. Protoc. Neurosci.* 8:27. 10.1002/0471142301.ns0827s57 21971850

[B24] Jhang J. Lee H. Kang M. S. Lee H. S. Park H. Han J. H. (2018). Anterior cingulate cortex and its input to the basolateral amygdala control innate fear response. *Nat. Commun.* 9:2744. 10.1038/s41467-018-05090-y 30013065 PMC6048069

[B25] Jones C. E. Monfils M. H. (2016). Dominance status predicts social fear transmission in laboratory rats. *Anim. Cogn.* 19 1051–1069. 10.1007/s10071-016-1013-2 27411940 PMC5054054

[B26] Karamihalev S. Brivio E. Flachskamm C. Stoffel R. Schmidt M. V. Chen A. (2020). Social dominance mediates behavioral adaptation to chronic stress in a sex-specific manner. *Elife* 9:e58723. 10.7554/eLife.58723 33034286 PMC7679136

[B27] Keum S. Shin H. S. (2016). Rodent models for studying empathy. *Neurobiol. Learn. Mem.* 135 22–26. 27475995 10.1016/j.nlm.2016.07.022

[B28] Keum S. Shin H. S. (2019). Neural Basis of Observational Fear Learning: a Potential Model of Affective Empathy. *Neuron* 104 78–86. 10.1016/j.neuron.2019.09.013 31600517

[B29] Kim A. Keum S. Shin H. S. (2019). Observational fear behavior in rodents as a model for empathy. *Genes Brain Behav.* 18:e12521. 10.1111/gbb.12521 30264490

[B30] Kim E. J. Kim E. S. Covey E. Kim J. J. (2010). Social transmission of fear in rats: the role of 22-kHz ultrasonic distress vocalization. *PLoS One* 5:e15077. 10.1371/journal.pone.0015077 21152023 PMC2995742

[B31] Kim S. Matyas F. Lee S. Acsady L. Shin H. S. (2012). Lateralization of observational fear learning at the cortical but not thalamic level in mice. *Proc. Natl. Acad. Sci. U.S.A.* 109 15497–15501. 22949656 10.1073/pnas.1213903109 PMC3458373

[B32] Knight E. L. Mehta P. H. (2017). Hierarchy stability moderates the effect of status on stress and performance in humans. *Proc. Natl. Acad. Sci. U.S.A.* 114 78–83. 27994160 10.1073/pnas.1609811114 PMC5224385

[B33] Labots M. Laarakker M. C. Ohl F. Van Lith H. A. (2016). Consomic mouse strain selection based on effect size measurement, statistical significance testing and integrated behavioral z-scoring: focus on anxiety-related behavior and locomotion. *BMC Genet.* 17:95. 10.1186/s12863-016-0411-4 27357390 PMC4928255

[B34] Labots M. M. Laarakker M. C. M. Schetters D. D. Arndt S. S. S. Van Lith H. A. H. (2018). An improved procedure for integrated behavioral z-scoring illustrated with modified Hole Board behavior of male inbred laboratory mice. *J. Neurosci. Methods* 293 375–388. 10.1016/j.jneumeth.2017.09.003 28939008

[B35] Langford D. J. Crager S. E. Shehzad Z. Smith S. B. Sotocinal S. G. Levenstadt J. S. (2006). Social modulation of pain as evidence for empathy in mice. *Science* 312 1967–1970. 10.1126/science.1128322 16809545

[B36] Larrieu T. Cherix A. Duque A. Rodrigues J. Lei H. Gruetter R. (2017). Hierarchical Status Predicts Behavioral Vulnerability and Nucleus Accumbens Metabolic Profile Following Chronic Social Defeat Stress. *Curr. Biol.* 27:2202–2210.e4. 10.1016/j.cub.2017.06.027 28712571

[B37] Ledoux J. E. (2000). Emotion circuits in the brain. *Annu. Rev. Neurosci.* 23 155–184. 10845062 10.1146/annurev.neuro.23.1.155

[B38] Liu H. Zhang C. Ji Y. Yang L. (2018). Biological and Psychological Perspectives of Resilience: is It Possible to Improve Stress Resistance? *Front. Hum. Neurosci.* 12:326. 10.3389/fnhum.2018.00326 30186127 PMC6110926

[B39] Masuda A. Aou S. (2009). Social transmission of avoidance behavior under situational change in learned and unlearned rats. *PLoS One* 4:e6794. 10.1371/journal.pone.0006794 19710921 PMC2728840

[B40] Olsson A. Phelps E. A. (2007). Social learning of fear. *Nat. Neurosci.* 10 1095–1102. 17726475 10.1038/nn1968

[B41] Panksepp J. B. Lahvis G. P. (2011). Rodent empathy and affective neuroscience. *Neurosci. Biobehav. Rev.* 35 1864–1875. 21672550 10.1016/j.neubiorev.2011.05.013 PMC3183383

[B42] Pisansky M. T. Hanson L. R. Gottesman I. I. Gewirtz J. C. (2017). Oxytocin enhances observational fear in mice. *Nat. Commun.* 8:2102. 10.1038/s41467-017-02279-5 PMC5727393 29235461

[B43] Ramos A. (2008). Animal models of anxiety: do I need multiple tests? *Trends Pharmacol. Sci.* 29 493–498. 18755516 10.1016/j.tips.2008.07.005

[B44] Ramos A. Pereira E. Martins G. C. Wehrmeister T. D. Izidio G. S. (2008). Integrating the open field, elevated plus maze and light/dark box to assess different types of emotional behaviors in one single trial. *Behav. Brain Res.* 193 277–288. 10.1016/j.bbr.2008.06.007 18590774

[B45] Rodriguez M. Ceric F. Murgas P. Harland B. Torrealba F. Contreras M. (2019). Interoceptive Insular Cortex Mediates Both Innate Fear and Contextual Threat Conditioning to Predator Odor. *Front. Behav. Neurosci.* 13:283. 10.3389/fnbeh.2019.00283 31998093 PMC6962178

[B46] Sapolsky R. M. (2005). The influence of social hierarchy on primate health. *Science* 308 648–652. 15860617 10.1126/science.1106477

[B47] Sial O. K. Warren B. L. Alcantara L. F. Parise E. M. Bolanos-Guzman C. A. (2016). Vicarious social defeat stress: bridging the gap between physical and emotional stress. *J. Neurosci. Methods* 258 94–103. 10.1016/j.jneumeth.2015.10.012 26545443 PMC4691556

[B48] Sillivan S. E. Joseph N. F. Jamieson S. King M. L. Chevere-Torres I. Fuentes I. (2017). Susceptibility and Resilience to Posttraumatic Stress Disorder-like Behaviors in Inbred Mice. *Biol. Psychiatry* 82 924–933. 28778658 10.1016/j.biopsych.2017.06.030 PMC5683920

[B49] Simpson A. J. Fowler S. J. Group U. B. S. (2018). Reclassification of Bronchodilator Reversibility in the U-BIOPRED Adult Asthma Cohort Using z Scores. *Chest* 153 1070–1072. 10.1016/j.chest.2017.10.051 29626951

[B50] Smith M. L. Asada N. Malenka R. C. (2021). Anterior cingulate inputs to nucleus accumbens control the social transfer of pain and analgesia. *Science* 371 153–159. 10.1126/science.abe3040 33414216 PMC7952019

[B51] Thirioux B. Harika-Germaneau G. Langbour N. Jaafari N. (2019). The Relation Between Empathy and Insight in Psychiatric Disorders: phenomenological, Etiological, and Neuro-Functional Mechanisms. *Front. Psychiatry* 10:966. 10.3389/fpsyt.2019.00966 32116810 PMC7020772

[B52] Twining R. C. Vantrease J. E. Love S. Padival M. Rosenkranz J. A. (2017). An intra-amygdala circuit specifically regulates social fear learning. *Nat. Neurosci.* 20 459–469. 10.1038/nn.4481 28114293 PMC5323274

[B53] Van Steensel F. J. A. Heeman E. J. (2017). Anxiety Levels in Children with Autism Spectrum Disorder: a Meta-Analysis. *J. Child Fam. Stud.* 26 1753–1767. 28680259 10.1007/s10826-017-0687-7 PMC5487760

[B54] Wang F. Kessels H. W. Hu H. (2014). The mouse that roared: neural mechanisms of social hierarchy. *Trends Neurosci.* 37 674–682. 10.1016/j.tins.2014.07.005 25160682

[B55] Wang F. Zhu J. Zhu H. Zhang Q. Lin Z. Hu H. (2011). Bidirectional control of social hierarchy by synaptic efficacy in medial prefrontal cortex. *Science* 334 693–697. 10.1126/science.1209951 21960531

[B56] Yehuda R. Hoge C. W. Mcfarlane A. C. Vermetten E. Lanius R. A. Nievergelt C. M. (2015). Post-traumatic stress disorder. *Nat. Rev. Dis. Primers* 1:15057. 10.1038/nrdp.2015.57 27189040

