# Supplementary figures and images for: A modified mouse model for observational fear learning and the influence of social hierarchy
Source: Front Behav Neurosci. 2022 Jul 25;16:941288. doi: 10.3389/fnbeh.2022.941288 (PMC9359141; doi:10.3389/fnbeh.2022.941288)

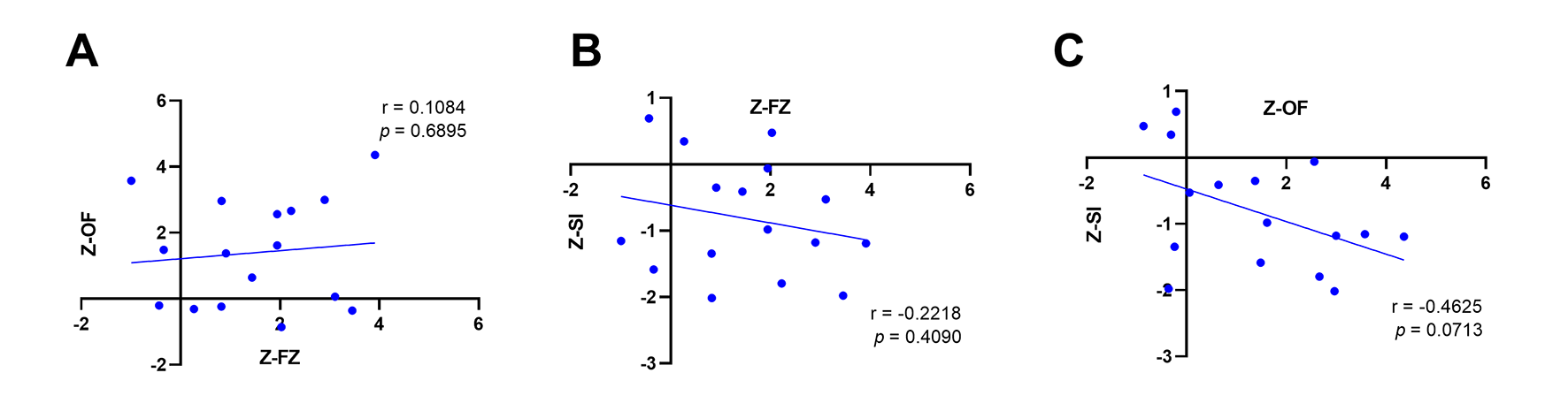

Supplement: Supplementary Figure 1 — The relationship with the integrated Z-scores of three different tests (FZ, OF, and SI) on observational fear training. (A–C) There were no significant correlations between the integrated Z-scores calculated from FZ, OF, and SI. Data are analyzed by Pearson’s correlation. [file Image_1.TIF]
